# Recent Advances in the Use of Metformin: Can Treating Diabetes Prevent Breast Cancer?

**DOI:** 10.1155/2015/548436

**Published:** 2015-03-19

**Authors:** Diana Hatoum, Eileen M. McGowan

**Affiliations:** ^1^School of Medical and Molecular Biosciences, Faculty of Science, University of Technology Sydney, Sydney, NSW 2007, Australia; ^2^School of Medicine, University of Sydney, Camperdown, Sydney, NSW 2006, Australia

## Abstract

There is substantial epidemiological evidence pointing to an increased incidence of breast cancer and morbidity in obese, prediabetic, and diabetic patients.* In vitro* studies strongly support metformin, a diabetic medication, in breast cancer therapy. Although metformin has been heralded as an exciting new breast cancer treatment, the principal consideration is whether metformin can be used as a generic treatment for all breast cancer types. Importantly, will metformin be useful as an inexpensive therapy for patients with comorbidity of diabetes and breast cancer? In general, meta-analyses of clinical trial data from retrospective studies in which metformin treatment has been used for patients with diabetes and breast cancer have a positive trend; nevertheless, the supporting clinical data outcomes remain inconclusive. The heterogeneity of breast cancer, confounded by comorbidity of disease in the elderly population, makes it difficult to determine the actual benefits of metformin therapy. Despite the questionable evidence available from observational clinical studies and meta-analyses, randomized phases I–III clinical trials are ongoing to test the efficacy of metformin for breast cancer. This special issue review will focus on recent research, highlighting* in vitro *research and retrospective observational clinical studies and current clinical trials on metformin action in breast cancer.

## 1. Introduction

Cancer and diabetes are two of the most common chronic diseases worldwide [1] with a strong association between the two diseases [2, 3]. Substantial evidence exists indicating that the risk of developing and dying from breast cancer is higher in diabetic patients compared to nondiabetic patients, excluding all other diseases [2]. Metformin, a biguanide oral antidiabetic drug, commonly used to treat type 2 diabetes mellitus has aroused much interest in comorbidity (diabetes/cancer) treatment, and emerging evidence from* in vitro* and epidemiological studies suggests that metformin improves the overall survival for cancer/diabetic comorbidity patients [2, 3].* In vitro* experimentation supports metformin as a strong candidate for treatment of breast cancer, where it has been shown to increase breast cancer cell death. However, the use of metformin as a comorbidity treatment, or breast cancer preventative therapy, in retrospective clinical meta-analyses studies is controversial. Metformin, on the one hand, has been shown to decrease cancer incidence and increase survival [3–6], while on the other hand no such association has been observed in other studies [7].

This special issue review brings together recent* in vitro* research supporting metformin as a wide-ranging treatment for most breast cancer subtypes, including the hard to treat triple negative subtype. Importantly, this paper will provide an overview of the recent contradicting meta-analyses and retrospective observational clinical studies focusing on metformin as a therapeutic agent for breast cancer.

### 1.1. Changing Metabolism Linking Diabetes and Cancer

For over a century, disturbances in cellular metabolism intrinsically linking diabetes and cancer have been recognized [8, 9]. One of the hallmarks of cancer is the reprogramming of energy metabolism to fuel cancer cell growth and division [10]. First proposed by Otto Warburg in 1924, cancer cells hijack cellular metabolism to favour aerobic glycolysis (high glucose demand) for energy needs in preference to mitochondrial oxidative phosphorylation [11]. Although aerobic glycosylation is an inefficient energy process, the bioenergetic demands of a cancer cell favour fast nutrients in the form of excess glucose for fast bursts of energy to fuel all the molecular components for DNA replication and cell division. Simplistically, prediabetes is inefficient processing of intracellular glucose, which leads to insulin resistant cells, hyperinsulinemia (increased insulin), and hyperglycemia (increased blood glucose levels). Elevated insulin levels have been shown to have mitogenic effects and constitute an increased risk factor for breast cancer [12]. An excessive supply of glucose in the bloodstream, as evidenced in diabetic patients, may provide the necessary nutrients to feed cancer cells; hence, the proposal that diabetic treatments reduce glucose in the bloodstream may prove beneficial for cancer prevention and patient therapy [13]. Metformin is commonly used in the treatment of type 2 diabetes mellitus to combat insulin resistance by reducing the amount of available glucose in the blood, as aptly described by Jalving and colleagues [13] “taking away the candy.” The antidiabetic drug metformin is emerging as a potential, efficient, preventative, and adjuvant therapy for many cancer types [14–17].

### 1.2. Safety of Metformin in Diabetic Treatment

For over than 50 years, metformin has been one of the most effective, well tolerated, antidiabetic treatments prescribed worldwide [18]. Metformin taken alone is a relatively safe drug for clinical use with only mild side effects documented including gastrointestinal disturbances (diarrhea, nausea, and irritation of the abdomen) [19]. The major toxicity reported is lactic acidosis, though this is very rare (9 per 100,000) [20]. A recent report suggests metformin is associated with impairment of cognitive function and these studies are ongoing [21]. The overall safety of metformin with minimal and rare side effects adds to its attractiveness as a potential breast cancer or comorbidity treatment for cancer patients with diabetes.

### 1.3. Diabetes and Breast Cancer

Evidence from epidemiological studies strongly supports that prediabetes, preexisting diabetes mellitus, and obesity are risk factors for cancer with a poorer outcome reported for breast cancers occurring in diabetic patients compared to nondiabetic patients [2, 22–26]. A meta-analysis of twenty clinical trials involving more than 1.9 million cancer patients with or without diabetes supported a significant increase in combined incidence and death from breast cancer [2]. This mega-study agreed with previous findings by Peairs and colleagues who reported comorbidity of breast cancer and diabetes was associated with a 49% increased risk of death from any cause and increased adverse effects in response to chemotherapy [24]. Prediabetes and hyperinsulinemia in breast cancer patients have also been associated with higher mortality rates [27–29]. Interestingly, a meta-analysis by Boyle and colleagues showed that the association between diabetes and breast cancer was restricted to diabetes mellitus type 2 (not type 1) in postmenopausal women and no such association was evident between diabetes and prediabetic conditions and breast cancer in premenopausal women [30]. The link between the onset of prediabetes, type 2 diabetes mellitus, and a higher risk of breast cancer diagnosis comes with new insights into how diabetic treatments influence breast cancer outcomes [2, 17, 24, 30]. Metformin, a well-tolerated insulin-sensitizer, has shown promise in reducing cancer risk or has no negative effect [29, 31–33]. Recently, mining of over 100,000 electronic medical records from Vanderbilt University Medical Center and Mayo Clinic by Xu and colleagues showed that the use of metformin significantly reduced cancer risk, including breast cancer, compared to patients who are not using metformin and are independent of diabetes status [6], thus providing additional support for metformin use in future cancer treatment regimens. Consequently, there has been much interest in understanding the mechanism of metformin action and exploring its efficacy in breast cancer therapy. Equally, there are a number of studies that do not support the observation of a reduction in breast cancer risk in diabetic and nondiabetic patients being treated with metformin and these findings are discussed.

In contrast, diabetic treatments, such as sulfonylureas, have been shown to increase mortality in patients with cancer and type 2 diabetes and insulin replacement has been shown to increase mortality due to its mitogenic effects [34–40]. However, it is noted that, in one meta-analysis retrospective study, data extracted from the Hong Kong Diabetes Registry reported that insulin replacement therapy reduced cancer risk [41]. An increase in body mass index (BMI) or obesity is associated with cancer risk and this study did not account for BMI [42]. Given the low BMI in the Asian population, this may contribute to the differences in the results [43].

## 2. Mechanism of Metformin Action to Inhibit Cancer

The exact molecular mechanism of metformin action is not clearly understood and has been hotly debated [44, 45]. Nevertheless, metformin action undisputedly has been shown to increase insulin sensitivity* in vivo*, resulting in reduced plasma glucose concentrations, increased glucose uptake, and decreased gluconeogenesis [46, 47]. High insulin levels are associated with increased breast cancer risk and poor patient survival outcome [17, 48]; therefore, metformin directly and indirectly reduces cancer cell proliferation through reduction of insulin levels and blood glucose levels. In the context of breast cancer risk, metformin has been shown to decrease circulating hormones such as androgen and estrogen where elevated levels are linked with postmenopausal breast cancer development [49, 50]. Thus metformin treatment may serve as a contributory factor in decreasing breast cancer risk.

**Figure 1:**
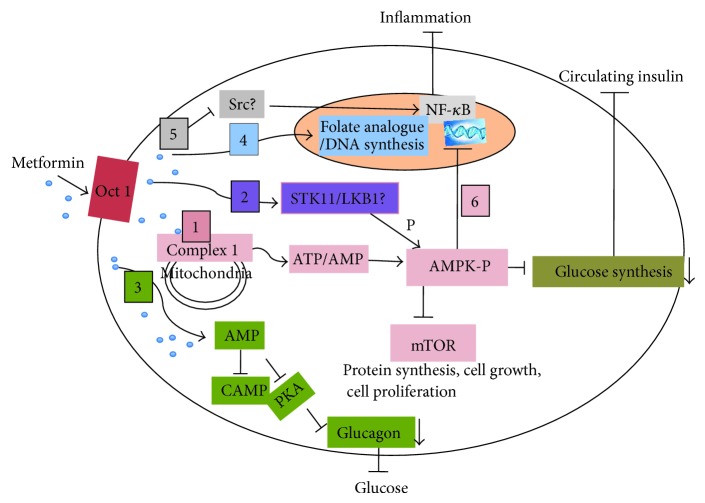
Mechanisms of metformin action to inhibit cancer. Metformin disrupts circulating glucose and insulin levels and reduces inflammation. The organic cation transporter (OCT1) mediates the first step in metformin cellular response [51–53]. (1) Metformin activates the AMPK-P pathway through inhibition of Complex 1 of the mitochondrial respiratory chain [54, 55]. This leads to the inhibition of mTOR and thus loss of cell proliferation and inhibition of glucose synthesis [56–59]. (2) LKB1 may act as an intermediatory of AMPK activation [60, 61]. (3) Metformin blocks cAMP and PKA, which in turn antagonizes glucagon action [62]. (4) Metformin acts as an antifolate hindering DNA replication [63]. (5) Metformin induces an anti-inflammatory response via the Src-mediated NF-*κΒ* pathway [64]. (6) Metformin action is implicated in both AMPK dependent and independent inhibition of the angiogenesis process [65].

The concept that cancer cells undergo metabolic reprogramming in favour of glycolysis is generally accepted. Metformin acts by interfering with cellular processes that facilitate insulin signalling and glucose synthesis. Some of these proposed signalling pathways are described in this section and illustrated in Figure 1.

There is general consensus that the organic cation transporter (OCT1) plays a major role in mediating the first step in metformin cellular response [51–53]. Shu and colleagues demonstrated that genetic variation in the OCT1 gene reduced hepatic uptake of metformin and altered the efficacy of metformin suggesting that patients with reduced response to metformin may be screened for OCT1 mutations [51]. The most widely accepted mechanism of metformin action is, by indirect activation of the central energy sensor, adenosine monophosphate-activated protein kinase (AMPK), which also plays a key role in insulin signalling [54, 55]. Activation of AMPK has been shown to inhibit the mammalian target of rapamycin (mTOR) and therefore inhibit pathological cell proliferation in different cancer cell lines [56–59] (Figure 1 (1)). Phosphorylation of AMPK by serine-threonine kinase 11/liver kinase B1 (STK11/LKB1) has also been reported to be an upstream event in metformin action [60, 61] despite more recent evidence questions whether LKB1 is required for metformin action [58, 66] (Figure 1 (2)). Whereas the focus of metformin action has been directed towards reduction of glucose synthesis through the AMPK pathway (Figure 1 (1)), Miller and colleagues showed that metformin antagonism of glucagon action was responsible for reducing fasting glucose levels [62] (Figure 1 (3)).

### 2.1. Metformin, Cancer, and the Mitochondria Conundrum

Upstream of AMPK-activation both mitochondria-dependent and -independent mechanisms have been described as precursors of AMPK activation. Metformin has been described as a “mitochondrial poison” through inhibition of Complex 1 of the mitochondrial respiratory chain leading to AMPK activation and reduction of glucose synthesis [54, 55] (Figure 1 (1)). Based on the premise that metformin is a weak “poison,” Salem and colleagues proposed that metformin could be useful as an anticancer therapy targeting mitochondrial metabolism [67]. Metformin also affects the mitochondrial redox state through inhibition of mitochondrial glycerophosphate dehydrogenase, which leads to suppression of gluconeogenesis [68]. These studies were confirmed in mouse and rat models using metformin treatment doses that achieved similar plasma concentrations to those observed in type 2 diabetes patients treated with metformin [68].

Alternatively, mitochondrial-independent AMPK activation has been described whereby metformin acts in a similar manner to an antifolate, a member of the antimetabolite class of chemotherapy drugs, and inhibits DNA replication and cell proliferation [63] (Figure 1 (4)).

Hirsch and colleagues implicated metformin in blocking the inflammatory response through inhibition of a step(s) in the Src-mediated-nuclear factor kappa B (NF-*κΒ*) signaling pathway [64] (Figure 1 (5)). These findings are especially relevant as a preventative measure in obesity-associated inflammation and cancer progression. Others have shown that metformin may be associated with inhibition of the angiogenesis process, as shown in endothelial cells, via AMPK-dependent and -independent pathways [65] (Figure 1 (6)). As new vascular formation is essential for tumour growth, this effect would assist in the prevention of cancer development.

In summary, metformin has been reported to have both direct and indirect effects on a number of metabolic pathways. Whilst the majority of laboratory research has focused on the mitochondrial-AMPK signalling pathway, new research has elucidated new mechanisms of metformin action, some of which are highlighted in Figure 1. Nonetheless, the mode of metformin action is still unclear and under investigation. The consensus is that the most important therapeutic endpoints of metformin are reduction in blood glucose level, and action as an insulin sensitizer, which is beneficial to patients with diabetes and/or potentially reduces the risk of most cancers including breast cancer.

## 3. Metformin and Breast Cancer* In Vitro* Studies

Since the benefits of metformin treatment for breast cancer patients were reported in 2005 [32], an increasing number of articles assessing its anticancer properties have been published. Highlighted here are some of the important findings from the* in vitro* studies linking metformin treatment and breast cancer outcome.

### 3.1. Breast Cancer Classification

Breast cancer is heterogeneous and, as such, different breast cancer subtypes are known to have distinct molecular profiles [69–74] and variable responses to different treatments. Based on the differential expression of various genes, breast cancer has been categorised into five major distinct molecular subtypes with prognostic significance: luminal A; luminal B; overexpression of HER2; also known as ErbB2; breast-like; and basal-like/triple negative [69]. Triple negative breast cancers have been further classified into six distinct subtypes: immunomodulatory, mesenchymal, mesenchymal stem-like, luminal androgen receptor, basal-like 1, and basal-like 2 [75]. In addition, there are at least seventeen rare subtypes defined [76]. Response to therapy is dependent on the pathology and classification of the breast tumour. The most predominant subtype, luminal A, is known to have the best prognosis with HER2 and the basal-like triple negative subtype has the worst outcome [77]. Nevertheless, many breast cancers recur and acquire resistance to conventional treatments. Metformin is being investigated* in vitro* in different breast cancer cell types, reviewed below, and an understanding of the mode of action in diverse breast cancer cell types is providing some insights into drug resistance. One of the leading questions is can metformin be used as a generic therapy for all breast cancer subtypes?

### 3.2. Metformin as Mono- or Combinational Therapy for Breast Cancer

There are enormous differences in clinical response to metformin monotherapy in diabetic and cancer patients; hence, the drug is generally used in combination with other treatments. The current challenge is to understand why this drug has reduced efficacy in some patients and to modify drug therapy for better outcome for individual patients. There have been a number of recent reports showing synergistic or enhanced effects on endpoints such as increased apoptosis and cell death in breast cancer cell lines when metformin is used in combination with chemotherapeutic drugs and with targeted therapies, providing a strong rationale for the use of metformin in clinical treatment regimens [78–80]. Metformin monotherapy has been shown to promote cell cycle arrest in both ER+ and ER− breast cancer cell lines [78, 80]. Metformin was reported to markedly suppress, but not completely abrogate, proliferation of breast cancer and cancer stem cells whilst being less toxic to normal stem cells [81]. These findings are important as a small proportion of breast cancer stem cells are believed to be the source of cancer recurrence [64]. Interestingly, cell cycle inhibition in a study by Lee and colleagues was significantly enhanced when the temperature was increased to 42°C suggesting that metformin may be more toxic to breast cancer patients with elevated body temperature [81]. In these experiments, metformin cytotoxicity appeared to be mediated through AMPK/mTOR activation [81].

### 3.3. Metformin Effects on Basal-Like/Triple Negative Breast Cancers

Triple negative breast cancers occur in a minority of breast cancer patients and such patients have a very poor prognosis [82]. These types of tumours are very aggressive and are associated with high morbidity and mortality due to their fast proliferation and propensity for metastasis. Their failure to express ER/PR and HER2 makes them resistant to antihormonal therapies and herceptin. Many triple negative breast tumours demonstrate epithelial mesenchymal transition (EMT) and stem cell-like properties and may lie dormant making them extremely difficult to treat with current chemotherapy treatments. Metformin has been shown to be a promising adjuvant treatment for triple negative breast cancers [58, 67, 78, 82–87] where Stat3 has been shown to be a critical regulator of metformin action [87], and it has also been shown to directly inhibit the enzymatic function of hexokinase I and hexokinase II [86]. However, not all studies have shown that metformin induces apoptosis and cell cycle arrest in the triple negative cell model, MDA-MB-231, and it has been suggested that this is a function of glucose homeostasis [58, 85].

### 3.4. Metformin Efficacy Is Dependent on Glucose Homeostasis

Circulating glucose levels may prove to be an important factor in response to metformin treatment in cancer patients. Menendez and colleagues reported that metformin lethality was enhanced in breast cancer cells that had undergone glucose deprivation [88]. Their studies showed metformin was protective in normal cells in the presence of physiological amounts of glucose, whereas it caused cell cycle arrest in breast cancer cells. Conversely, withdrawal of glucose induced breast cancer cell death independent of the following subtypes: ER+, HER2+, and triple negative [88]. Further studies have also confirmed that the failure to maintain glucose homeostasis results in a more aggressive triple negative breast cancer phenotype [85]. Moreover, in hyperglycemic conditions, Zordoky and colleagues showed that a surplus of glucose supply rescued the triple negative MDA-MD-231 cells from metformin induced cell death and suggested that the bypass was due to the generation of enough energy for proliferation through aerobic glycolysis using the excess glucose [58]. Based on the laboratory evidence, it has been advocated that glucose monitoring of breast cancer patients may provide some insight into patient response to metformin and that pharmacological deprivation of glucose combined with metformin treatment may benefit patients with high glucose levels [88].

### 3.5. Use of Metformin to Overcome Multidrug and Chemotherapy Resistance in Breast Cancer Cells

The emergence of multidrug/chemotherapy resistant cells within a tumour population is a major obstacle for many cancer patients. There is now compelling evidence to suggest that metformin resensitizes cells and cooperates with some anticancer drugs to improve efficacy through reprogramming of the metabolic cellular pathways [89, 90]. A recent study showed the reversal of multidrug resistance in breast cancer cells through activation of AMPK/mTOR by metformin [90]. In addition, metformin promoted 5-FU-induced apoptosis, consistent with its proposed role as a pseudo metabolite, and reversed epithelial mesenchymal transition (EMT), a critical phenotypic switch associated with enhanced capacity of cells for invasion, metastasis, and chemoresistance [90]. Metformin sensitisation to chemotherapy has also been demonstrated in breast cancer cells overexpressing aldehyde dehydrogenase (ALDH), an enzyme linked to chemoresistance in breast cancer cells that also feature an EMT phenotype [91]. Potentially, small doses of metformin could be used as an adjuvant therapy to prevent some chemotherapy resistant phenotypes and prevent EMT transition.

ErbB2-positive (HER2/neu) breast cancer cells are usually treated with lapatinib (a dual inhibitor of the EGRF and ERBB2/HER2 tyrosine kinase inhibitor) as a first line monotherapy [92–94]. Short-lived clinical responses in ErbB+ breast cancers are due to acquired resistance to lapatinib. Komurov and colleagues showed that forcing ErbB2 drug-sensitive cells into glucose-deprivation made them more resistant to lapatinib [95]. In line with the glucose-deprivation concept described above, metformin counteracted lapatinib-induced toxicity [95]. Combinational therapy of metformin and conventional chemotherapy treatment, such as carboplatin, doxorubicin, and paclitaxel, were shown to contribute to synergistic inhibition of cell proliferation in most breast cancer cell types [78]. The use of metformin to counteract or prevent tamoxifen resistance has also been explored in breast cancer cell lines with positive results. The combination of tamoxifen and metformin has been shown to augment the apoptotic effect of tamoxifen alone [79, 96]. As demonstrated, metformin-induced alteration in cancer cell metabolism appears to be an effective adjuvant therapy for many different types of chemoresistant breast tumours.

### 3.6. Metformin Failure in Prevention and Treatment of Breast Cancer

Resistance to treatment is inherent in breast cancer and metformin is proving to be no exception. The Menendez group used chronic metformin exposure to establish metformin resistant cells [97]. Acquired metformin resistance triggered a transcriptome reprogramming event in breast cancer cells and the cells developed a highly metastatic stem-like expression profile making these cancer cells more difficult to treat [97]. Metformin efficacy was also reduced in breast cancers overexpressing BCA2, a gene associated with an AMPK-suppressive function [98]. The BCA2 gene is overexpressed in >50% of breast cancer patients making it a potential target/adjuvant therapy for metformin resistant breast cancer cells [98]. These studies advocate an individualized genetic approach targeting specific genetic mutations, such as BCA2, with combinational treatment to reduce acquired resistance to metformin.

In summary, the heterogeneous nature of breast cancer makes the disease difficult to treat. However,* in vitro* studies strongly support a role for metformin, which is one of the most commonly used diabetic medications, as a generic therapy for most, if not all, breast cancer subtypes. Furthermore, the potential to use metformin as a dual treatment for cancer and diabetes is an important consideration with the increasing incidence of comorbidity worldwide. As highlighted in these* in vitro* studies, the mechanism of metformin action is still unclear and affects more than one cellular signaling pathway. Breast cancer is inherent and acquired resistance to metformin is still to be explored.

## 4. Breast Cancer Retrospective Observational Clinical Studies

*In vitro* studies examining the use of metformin as a breast cancer therapy for most breast cancer subtypes have been very promising; however, translating these positive findings into reduced breast cancer incidence and improved clinical outcomes with metformin use has come with very mixed and contradictory reviews.  Table 1 summarises the important points arising from the recent meta-analyses as highlighted in this section.

The subtype of breast cancer, the presence or absence of hormones and hormone receptors; the age of the patient (pre- or postmenopausal); comorbidities, such as prediabetes, diabetes, and other diseases; and comorbidity treatments all impact on the efficacy of relapse-free survival (RDFS), metastasis-free survival (MDFS), and patient overall disease-free survival (OS). The majority of breast cancers are present in postmenopausal women where there is a higher risk of comorbidity with diabetes, obesity, and other age-related diseases. A number of meta-analyses of clinical study data support the use of metformin as a breast cancer adjuvant treatment with improved patient outcome in postmenopausal women [22, 23, 99, 114]. A study by Currie and colleagues showed that mortality increased in elderly breast cancer patients with diabetes, and metformin treatment improved survival rates in comparison with other diabetic treatments (sulfonylureas and insulin) and compared to a nondiabetic patient cohort [29]. In agreement with these findings, a study by Kiderlen and colleagues showed that metformin increased the RDFS in elderly breast cancer patients with diabetes compared to nondiabetic patients, with no difference between patients with other comorbidity diseases [100]. In a retrospective clinical meta-analysis of 28 separate studies by Zhang and Li, they found that, in breast cancer patients with existing diabetes, metformin reduced the mortality of breast cancer and reduced the risk of breast cancer by 6% [3]. In addition, elderly nondiabetic breast cancer patients had similar survival rates to diabetic breast cancer patients using metformin with elderly diabetic patients treated with metformin having a higher RFS period [100]. Metformin was also associated with reduced incidence of invasive breast cancer in postmenopausal women [99].

*Can Metformin Be Used as a Generic Therapy for All Breast Cancer Sub-Types?* Xiao and colleagues looked at specific breast cancer subtypes and found that the nondiabetic metformin group of patients with Luminal A (ER+/PR+), Luminal B (high ki67), and luminal B (HER-2/NUE+) had better prognosis compared to the nondiabetic group not treated with metformin. However, in diabetic groups, only luminal A and luminal B (HER-2/NUE+) metformin treated patients had better prognosis than nonmetformin group [101]. Concurring with these findings, in independent studies metformin showed decreased cell proliferation in insulin resistant, luminal B subtype breast cancer patients although overall metformin did not significantly alter cell proliferation in this patient cohort [102] and diabetic patients with HER2+ subtype had a better prognosis with metformin [115]. In contrast, looking at patients' data from 2005 to 2011, Besic and colleagues indicated that the long-term use of metformin in diabetic breast cancer patients does not associate with breast cancer subtype distribution [103]. Berstein and colleagues showed that postmenopausal diabetic breast cancer patients treated with metformin as a monotherapy or metformin and sulfonylurea were found to have higher progesterone receptor (PR) tumours than patients treated with other antidiabetic therapies leading to better response of these breast cancer patients to hormone therapy [104]. In contrast, Besic's group found that there was no change in the rate of PR between metformin and nonmetformin groups [103]. In this study, 253 patients (both pre- and postmenopausal patients) were reviewed.

Although the studies described showed metformin to have tantalizing promise as a comorbidity treatment for cancer patients with diabetes and treatment for breast cancer subtypes, most of these studies were inconclusive.

Not all meta-analyses reports showed a positive correlation with improved patient mortality and metformin treatment. Five recent reports, one comprised of a meta-analysis of twenty-one observational studies subgrouped by cancer type, did not show any significant reduction in mortality in breast cancer patients [105–107, 116, 117]. Although there have been very promising* in vitro* studies for the use of metformin in triple negative breast cancer therapy, these reports have not been confirmed in clinical observational studies where no significant impact on survival outcome has been observed, even though there was a trend towards reduced distant metastasis in these cohorts [108]. There are a number of examples where* in vitro* data did not correlate with clinical observations. Samarajeewa and colleagues found metformin specifically inhibited aromatase expression* in vitro* [118], whereas Bershtein's group found that this did not translate to clinical samples where they observed that metformin did not inhibit aromatase expression in tumour samples from diabetic breast cancer patients [119]. As metformin is a well-tolerated drug for diabetes with very few side effects, the important question is that can we continue to use this drug in combination with traditional cancer therapies for comorbidity patients? One study by Ferro and colleagues showed that metformin caused radiotoxicity in breast cancer patients with diabetes compared to nondiabetic patients and diabetic patients receiving alternative medications [109]. With the increasing comorbidity of breast cancer with diabetes and other diseases in postmenopausal women, combination comorbidity medication studies are imperative to determine metformin interactive efficacy.

### 4.1. Metformin as a Breast Cancer Treatment Independent of Diabetes

Despite the benefits of metformin to reduce breast cancer risk in diabetic patients metformin is still debatable; metformin is coming into prominence in its own right as a breast cancer adjuvant treatment independent of diabetes. As mentioned earlier, in addition to metformin's properties to reduce glucose and insulin in the bloodstream, it has also been shown to reduce circulating androgen and estrogen levels, which have well established mitogenic effects in breast cancer [49, 50]. Endocrine resistant breast cancer in obese postmenopausal women is partly mediated by insulin resistance and changes in estrogen metabolism metformin may also play a crucial role in preventing endocrine resistant tumours. However, early Phase I clinical trials with a combination of metformin with exemestane, an aromatase inhibitor, in a cohort of obese nondiabetic postmenopausal women, though well tolerated, showed no improved outcome [120]. A prospective phase II clinical trial to test neoadjuvant metformin with the aromatase inhibitor letrozole in ER+ postmenopausal nondiabetic women has been initiated to evaluate the direct antitumour effects of metformin [110] and it will be some time before the long-term benefits of metformin use is realised.

### 4.2. Metformin Presurgical Trials in Breast Cancer Patients without Diabetes

Four presurgical metformin clinical trials to determine if metformin was able to modulate breast tumour proliferation were conducted with mixed results. Three clinical trials showed no significant difference in apoptosis when metformin was given before the surgery [102, 111]; conversely, one trial indicated a potential benefit according to insulin-resistant status [112] and one trial provided support for antiproliferative effects with metformin [113]. The major limitation to of all these studies was the small sample size.

Despite the controversial retrospective meta-analyses studies reported, currently there are at least 20 recruiting and completed clinical trials, registered by the National Institute of Health (NIH) USA, addressing the use of metformin with combinational cancer therapies (https://clinicaltrials.gov/ct2/results?term=breast+cancer+and+metformin&Search=Search). To date, the results of one study have been posted on the NIH clinical trials site; however, due to the low numbers in the patient cohorts, no meaningful results have been recorded.

## 5. Conclusion

Overwhelming evidence supporting that type 2 diabetes increases breast cancer risk makes the idea of using the diabetic drug metformin as a preventative drug for cancer a very exciting prospect. Still there are a number of unresolved issues in metformin use for breast cancer treatment outlined as follows.

*Summary. Metformin Use for Breast Cancer Treatment*. There is strong epidemiological evidence to support an increase in breast cancer incidence and death in prediabetic and diabetic patients.

There is mounting evidence to suggest that diabetic patients treated with metformin have reduced breast cancer risk supporting metformin use as a preventative medication for breast cancer.

*In vitro* studies strongly support the role for metformin in treatment for most of, if not all of, the subtypes of breast cancer, especially the hard to treat triple negative breast cancers.

To date, meta-analyses of retrospective clinical trial data on the use of metformin as a mono- or combined therapy for comorbidity (patients with diabetes and cancer) are equivocal supporting positive or no difference in survival outcomes. Most studies are inconclusive and recommend further confirmation.

Phase I clinical trials with a combination of metformin with exemestane in a cohort of obese, nondiabetic postmenopausal women, although well tolerated, showed no improved outcome.

The majority of breast cancers patients are postmenopausal women where there is increasing incidence of comorbidity, diabetes, and cancer. The heterogeneity of breast cancer, confounded by comorbidity of disease in the elderly population, makes it difficult to determine the actual benefits of metformin as a mono- or adjuvant therapy for breast cancer.

Prospective controlled clinical trial outcomes will be important to provide more definitive answers regarding the efficacy of metformin use in prevention and treatment for a breast cancer. Ongoing clinical trials are open for metformin as an adjuvant therapy for breast cancer.

The biology and mechanism of metformin action underpinning its use as an antidiabetic and antibreast cancer comorbidity treatment are likewise very compelling. Although the mechanism of metformin action is not fully understood, the* in vitro* evidence shows that metformin is an effective inhibitor of cell proliferation and an activator of apoptosis in breast cancer cells and supports the use of metformin as a mono- and/or adjuvant therapy for breast cancer with some limitations as discussed. Data from the retrospective meta-analyses investigating the use of metformin in breast cancer have suffered from a number of limitations and flawed assumptions. The meta-analyses are retrospective observational studies only and were not designed to specifically analyse the effects of metformin as a preventative or adjuvant treatment in defined breast cancer patient cohorts. Patient numbers and confounding comorbidities limited many of the studies. The majority of the studies report a significant increase in breast cancer incidence in postmenopausal type 2 diabetic, prediabetic, and obese patients with higher prevalence of other comorbidities such as cardiovascular disease. Retrospective studies to investigate if the use of metformin as a preferred diabetic medication actually reduced the incidence of breast cancer in these population studies have been contentious and divided into some meta-analyses showing a decreased risk of breast cancer incidence and others showing no effect. Other aspects that can alter patient outcome after metformin treatment include other medications taken, the different administration times of taking the drugs, and the drug dosage. These need to be taken into account in future studies. To date, metformin is not approved for clinical use in breast cancer treatment by the Food and Drugs Administration (FDA) and is still considered investigational. Even so, metformin is well established as an inexpensive, relatively safe, and effective drug for diabetes, prediabetes, and obesity and to extend this into breast cancer treatment regimens may have both economic and clinical benefits. Two important issues that are still to be resolved are the safety of metformin in comorbidity treatments for breast cancer and diabetes and the suitability of metformin as a breast cancer therapy independent of diabetes. The persuasive* in vitro* evidence and the optimistic retrospective observational clinical meta-analyses studies on metformin treatment for breast cancer have led to ongoing phases I–III clinical trials. These studies are important for clarification of the use of metformin in breast cancer prevention and breast cancer treatment, particularly as it is a commonly used FDA approved drug for diabetes. Prospective controlled clinical trial outcomes will be important to provide more definitive answers regarding the efficacy of metformin use in prevention and treatment for a breast cancer patients as well as its efficacy in comorbidity treatments for diabetes, breast cancer, and other diseases.

## Figures and Tables

**Table 1:** BCa outcome with metformin treatment with/without diabetes mellitus.

References	Patient cohort	Age group	Ethnicity	BCa type	Key findings
Currie et al. 2012 [29]	112408 (8392 DM) (i) 24393 BCa (no DM) (ii) 1182 BCa (with DM)	>35 years	N/S	N/S	No mortality differences with/without metformin at diagnosis

Chlebwoski et al. 2012 [99]	68,019 patients (3401 DM) (i) 3273 invasive BCa	Postmenopausal	Mixed race	ER+ PR+ HER2+ HER2− Noninvasive Invasive	Women with diabetes were older and were more likely to be black and obese. Women with diabetes on metformin had a reduced BCa risk: Associated with PR+ ER+ Associated with HER2+? No association with HER2− Lower incidence of invasive BCa

Kiderlen et al. 2013 [100]	3124 (505 DM) (i) nonmetastatic BCa	Postmenopausal	N/S (Netherlands)	ER/PR+ ER/PR− Not defined	Patients with diabetes had overall better relapse-free survival (possibly through the effect of metformin, speculated not proven)

Xiao et al. 2014 [101]	5785 Luminal-type BCa (680 DM) (i) 1384 luminal A (201 diabetes) (ii) 3393 luminal B, high Ki67+ (341 DM) (iii) 1008 luminal B (her-2/neu+) (138 DM)	Pre-and postmenopausal	Asian	Luminal A Luminal B (Ki67) Luminal B (HER2+)	BMI was not a prognostic factor in these studies Metformin versus nonmetformin: Better prognosis for all subtypes Compared to metformin group, risk of death was higher in nonmetformin group. No significant difference between metformin and control groups. Diabetic patients: Metformin better prognosis for Luminal A and Luminal B (HER2+) Metformin poorer prognosis for Luminal B (high Ki67)

Bonanni et al. 2012 [102]	200 (non-DM) (i) 100 metformin (ii) 100 placebo	>18 years both pre and postmenopausal	N/S (Milan)	Luminal A Luminal B (Ki67) Luminal B (HER2+) TNBC	Metformin treatment: Overall, no change in Ki67 Overall, positive effect on insulin resistance Luminal B tumours-trend, decreased proliferation. Overweight or obese-trend, decreased proliferation

Besic et al. 2014 [103]	573 (invasive BCa) (i) 253 patients (+DM) (ii) 128 + metformin (iii) 125 no metformin. (iv) 320 BCa (no DM)	38–93 years (median age, 67)	N/S (Slovenia)	Luminal A Luminal B HER2 TNBC	DM + metformin—lower grade BCa compared to no metformin No change in ER/PR status with metformin Noted: Long-term metformin treatment was correlated with different BCa subtype distribution

Berstein et al. 2011 [104]	90 (BCa and DM)	48–82 years postmenopausal	N/S	ER+ PR+	Metformin increased PR in diabetic patients. Potentially increasing endocrine therapy success

Lega et al. 2014 [105]	Meta-analyses—Cancer patients with diabetes (all cancer types)	All ages	N/S	All types	No correlation between BCa and metformin and increased survival

Lega et al. 2013 [106]	2361 (BCa and DM)	>66 years	Ontario Ethnicity N/S	All types	No significant reduction in mortality or DFS in patients using metformin

Oppong et al. 2014 [107]	2889 (BCa + chemotherapy) (i) 141 (BCa + DM) (ii) 104 (DM at BCa diagnosis) (iii) 37 (DM + BCa diagnosed 6 mth)	38–80 years Majority Postmenopausal	Caucasian (72) African/American (52), Asian (10), Hispanic (4)	ER+, ER− PR+, PR− HER2+, HER2−	No difference between metformin and nonmetformin users in RFS, OS, and contralateral BCa

Bayraktar et al. 2012 [108]	1448 (triple negative BCa—TNBC) (i) 63 diabetic + metformin (ii) 67 diabetic no metformin (iii) 1318 non diabetic	More diabetic patients were postmenopausal	black, and obese	Triple negative	Metformin does not significantly impact on survival in TNBC. Trend toward decreased risk of distant metastasis in DM patients receiving metformin compared to non-DM

Ferro et al. 2013 [109]	110 (BCa) (i) 51 + metformin (DM) (ii) 28 no metformin (DM) (iii) 51 non-DM	>50	Mixed (white, black, other)	All types	Radiation therapy and metformin treatment Metformin associated with increased local radiation toxicity compared to nonmetformin users

Kim et al. 2014 [110]	208 (BCa—no DM) (i) 104 + Letrozole (ii) 104 + letrozole + metformin	Postmenopausal	Asian (Korean)	ER+	Study in progress

Kalinsky et al. 2014 [111]	33 non DM patients (Obese) (i) 9 DCIS (ii) 24 invasive BCa	>21 years	80% Hispanic	85% HR+ 20% triple negative	Metformin treatment pre-surgery—No reduction in proliferation of BCa tumour. Reduction in diabetic markers (insulin resistance)

Cazzaniga et al. 2013 [112]	100 BCa patients-Analysed (i) 45 metformin (ii) 42 placebo.	45–62 years	N/S (Milan)	Luminal A Luminal B Her2+ Triple negative	Metformin treatment pre-surgery—No reduction in proliferation of BCa tumour. Reduction in diabetic markers (insulin resistance)

Hadad et al. 2011 [113]	55 (BCa—no DM) (i) 25 completed met. (ii) 22 no metformin	41–82 years Pre- and postmenopausal	N/S	N/S	This trial supports antiproliferative effects of metformin in BC patients

BCa: breast cancer; DM: diabetes mellitus; N/S: not stated; TNBC: triple negative BCa.
